# The Fractionalization and Anthropocentric View of Comparative Psychology. Commentary: A Crisis in Comparative Psychology: Where Have All the Undergraduates Gone?

**DOI:** 10.3389/fpsyg.2015.01753

**Published:** 2015-11-17

**Authors:** Murray R. Horne, Cameron A. Ryczek

**Affiliations:** Psychology Department, California State University, East BayHayward, CA, USA

**Keywords:** comparative psychology, education, undergraduate, recruitment, comparative cognition

Comparative psychology is dead! Or at least may be waning if we do not recruit capable undergraduates. We must disagree with this prophecy that Abramson (2015) warns us about. The controversial nature of his opinion on the state of comparative psychology as a discipline lies in how one perceives the fractionalization of the field and the importance of connecting animal to human behavior. The key to understanding the current state of comparative psychology is not to take the approach that it is a dying field, but to understand how the field can further evolve.

According to Abramson (2015) the division of the discipline into multiple sub areas, including but not limited to comparative cognition, ethology, biopsychology, and sociobiology, distracts from what was unique about comparative psychology. Graduate students of comparative psychology were traditionally trained using an interdisciplinary approach, getting the breadth of training necessary to perform comparative analysis in humans and animals. Now graduate students are getting more specialized training. For instance, students in our lab get trained in spatial and associative learning in rodents, with little to no direct exposure to human research or any other animal species. This can be interpreted in two ways. First, the discipline of comparative psychology is hanging on by a thread. As soon as the last great comparative psychologist of our time passes on, there will be a collapse of comparative psychology as we know it. Second, this increase in specialization is demonstrative of scientific progress. One would not argue that the specializations of developmental, cognitive, or neuroscience takes anything away from what it means to be a psychologist, or that psychology as a discipline is at risk of fading away. The same is true for comparative psychology. The recent advancements of genetics (e.g., genomic sequencing, epigenetics) and neuroscience (e.g., small animal functional imaging techniques) have provided vast knowledge that comparative psychologists use. Thus the increase in specialization in the field is not something that should be repressed, but rather welcomed. We should not look to be changing the field's current status, but should be considering further developments to improve standing perceptions. Comparative research will still maintain its long tradition of being interdisciplinary, but the research will be conducted differently, with an increase in collaboration across academic departments that will escalate the production of research and foster a stronger research community.

Another point that Abramson (2015) argues strongly is that there should be an emphasis on the importance of connecting animal to human behavior. On one hand, this would potentially recruit more students who are interested in an applied field of research. On the other hand, Dewsbury (1984) suggested that making generalizations from animal to human was not appropriate for a “true” comparative psychologist. Rather, comparative psychologists study animals that are phylogenetically related and study those animals on a range of behaviors. For instance, Kamil et al. (1994) studied spatial learning in four related seed-caching corvids. The results provided insights on the importance of natural history and hippocampus volume for performance on a spatial task. The anthropocentric view taken by Abramson (2015) should be discouraged because it diminishes the importance of basic research, and discredits any foundational discovery; neglecting the possibility for further scientific advancement (Shettleworth, 1993, 2010). Curiosity should drive research and there is something to be said for gaining new knowledge for the sake of knowledge. I am not inferring that we should not be applying our research to human behavior or real world problems, but one needs to be aware that if we restrict research to just those that have a direct relationship to humans, then you undoubtedly restrict the discipline. This ultimately may result in the same worry that Abramson expresses, that comparative psychology is soon to be non-existent.

It is hard to argue with Abramson (2015) on the problems and the ameliorations of recruiting students to the discipline. It is true that students have a poor understanding of what comparative psychology encompasses because it is such a large area with many interconnecting disciplines. I agree that more education of the students is necessary, starting with arming undergraduates with an abundance of resources surrounding various courses and their respective learning outcomes. In addition, more education should be provided to academic advisors and career counselors to ensure that students who are interested in comparative research get appropriate feedback when selecting courses and for possible career opportunities. However, I think us as researchers have the most impact of recruiting students. Galef (1987, p. 260) sums it up the best, “Exciting research will do more for the future of the discipline than revisionist histories or calls for return to the true faith.”

We think it is interesting that Galef (1987) gave his opinion of the state of the discipline 28 years ago and we are continuing that debate with many common themes. Just knowing this should give some relief that comparative psychology is a robust discipline that withstands time. There is little to worry about the state of comparative psychology. Twenty years from now, we will probably be having this same debate with the new and younger generation of comparative psychologists. We therefore must proclaim: Long live comparative psychology… or whatever you want to call the discipline.

## Author contributions

MH and CR contributed equally to the opinions expressed in the current commentary.

### Conflict of interest statement

The authors declare that the research was conducted in the absence of any commercial or financial relationships that could be construed as a potential conflict of interest.
